# Phase II trial of sequential gefitinib after minor response or partial response to chemotherapy in Chinese patients with advanced non-small-cell lung cancer

**DOI:** 10.1186/1471-2407-6-288

**Published:** 2006-12-16

**Authors:** Jian Ming Xu, Yu Han, Yue Min Li, Chuan Hua Zhao, Yan Wang, Angelo Paradiso

**Affiliations:** 1 Beijing 307 Hospital Cancer Center, Beijing, China; 2 National Cancer Institute, Bari, Italy

## Abstract

**Background:**

Basic research of gefitinib (Iressa, ZD1839) has demonstrated the combination effects of gefitinib and chemotherapy were sequence-dependent. To evaluate the efficacy of sequential administration of gefitinib following a minor response or partial response to two to three cycles of chemotherapy, a phase II clinical trial was done in Chinese patients with advanced non-small-cell lung cancer (NSCLC).

**Methods:**

Thirty-three consecutive patients with advanced NSCLC that had been pretreated with at least one chemotherapeutic regimen and were responding to chemotherapy following 2 to 3 cycles of treatment, entered the trial from May 2004 to February 2006. Patients received gefitinib at an oral dose of 250 mg once daily for 4 weeks.

**Results:**

Thirty-three patients were evaluable for response and toxicity. The objective response rate was 24.2% (8 of 33)(95% CI, 11% to 42%). The symptom improvement rate was 54.5% (18 of 33) (95% CI, 41% to 69%). The median duration of response was 7 months (95%CI, 4.0 to 13.2 months). The median time to disease progression (TTP) was 6.5 months (95%CI, 0.7 to 16.6 months). The median overall survival time (OS) was 9.8 months (range, 2.1 to 18.0 months), and the actuarial 1-year survival was 36.4%. Toxicity was relatively mild and included only one patient (3.0%) with grade 4 diarrhea, 1 (3.0%) with grade 3 rash, 1 (3.0%) with grade 3 nausea, and 1 with grade 3 vomiting (3.0%).

**Conclusion:**

Preliminary results suggest that sequential administration of gefitinib following a response to chemotherapy may be beneficial for Chinese patients with advanced NSCLC. Further randomized clinical trials are needed.

## Background

Despite the use of platinum-based combination chemotherapy, which has shown to prolong survival in patients with advanced non-small-cell-lung cancer (NSCLC) when compared to best supportive care, the prognosis of advanced NSCLC continues to be poor with a median survival of about 10 months [1,2]. The goals of chemotherapy are to palliate symptoms, improve quality of life (QOL), and prolong survival. Treatment-related toxicities resulting in impaired QOL are major concerns for combination chemotherapy [3,4]. Second line treatment options, including docetaxel, pemetrexed, and erlotinib, have been approved in the United States for patients who failed to platinum-based chemotherapy, but they have limited efficacy and potentially considerable toxicity [5,6]. More treatment options are needed for patients with advanced NSCLC.

Gefitinib (Iressa, AstraZeneca, Alderley Park, Macclesfield, United Kingdom) is a synthetic anilinoquinazoline compound that inhibits epidermal growth factor receptor (EGFR) tyrosine kinase *in vitro *at nanomolar concentrations [7]. Several clinical studies have demonstrated the antitumor activity of gefitinib, with response rates ranging from 9% to 20% even in the second and the third line settings [8,9]. Impressively, improvement in tumor-related symptoms was observed in nearly 40% of patients, and often within days of starting gefitinib therapy [8-10]. Two combination trials with gefitinib, INTACT 1 and 2, counting over 2,000 enrolled patients, failed to demonstrate any improvement in overall survival (OS), time to disease progression (TTP), and response rates (RR) with the gefitinib combination compared to standard chemotherapy [11,12]. However, previous preclinical studies had demonstrated that concomitant administration of gefitinib and chemotherapy could render tumor cells less sensitive to chemotherapy, whereas, sequential regimens with gefitinib administration following chemotherapeutic agents, such as oxaliplatin or CPT-11, enhanced and maintained cell damage from chemotherapy [13-15]. Based on our clinical experience, response is usually apparent after the initial two to three cycles of chemotherapy.

Therefore, the present open-labeled, nonrandomized phase II trial was performed to evaluate the clinical efficacy and toxicity of sequential administration of gefitinib following a minor response or partial response to chemotherapy in advanced NSCLC.

## Methods

### Patient eligibility

All the patients enrolled in this study had histologically confirmed, measurable, locally advanced or metastatic NSCLC. Measurable disease had to be completely outside the radiation portal. Patients with stable brain metastases were eligible. Different chemotherapy regimens were allowed, as long as a minor response (MR), or partial response (PR) was obtained after two to three cycles of the last chemotherapeutic regimen. Gefitinib was started within 3 days of the response evaluation for the last cycle of chemotherapy. Patients were older than 18 years of age, and had an Eastern Cooperative Oncology Group (ECOG) performance status (PS) of 0 to 3, a life expectancy of 12 weeks or longer, a WBC count ≥ 3.0 × 10^9^/L, platelet count ≥ 100 × 10^9^/L, bilirubin less than 1.5-fold of the upper limit of normal (ULN), ALT or AST less than three-fold of the upper limit of institutional reference value (elevated to five-fold of the ULN in patients with known hepatic metastases), and a calculated creatinine clearance rate of more than 45 mL/min. Patients with a history of other malignancies were not eligible for treatment. All patients provided written informed consent before chemotherapy, and trial document approval was obtained from the ethics committee before study registration.

### Treatment plan

Gefitinib was administered at 250 mg once daily in the morning or afternoon, at approximately the same time, and continued until disease progression. Before initiation of treatment, all the patients underwent a complete physical examination, their medical history was taken and blood count, serum biochemistry tests (hepatic and renal function tests and electrolytes), urinalysis, and echocardiogram performed; a chest x-ray and computed tomography (CT) or magnetic resonance imaging (MRI) scans of all disease sites were obtained during the 3 weeks prior to study entry. Systemic anticancer therapy was not permitted during the trial, except for palliative radiotherapy in patients with isolated symptomatic bone metastases.

### Treatment assessment

Disease staging and response were assessed by clinical examination, chest x-ray, and computed tomographic (CT) scans. Bidimensionally measurable disease was evaluated by imaging procedures (chest x-ray, ultrasonography, CT scanning, magnetic resonance imaging). Response assessments were performed every 4 weeks and confirmed by a repeat measurement no less than 4 weeks from the first claim of a response. We assessed objective tumor response as complete response (CR), partial response (PR), stable disease (SD), or progressive disease (PD) in accordance with the standard WHO response criteria [16]. TTP was defined as the interval from initiation of gefitinib to disease progression, and OS was defined as the interval from initiation of responding chemotherapy until death.

Disease-related symptom improvement was measured using the Lung Cancer Subscale (LCS), a validated subscale of the QOL instrument, of the Functional Assessment of Cancer Therapy-Lung (FACT-L) questionnaire [17].

Toxicities were graded according to the National Cancer Institute Common Toxicity Criteria (NCI-CTC) version 2.0 before each therapy course.

### Statistical analysis

The objective response rate was determined with 95% confidence intervals. TTP, and OS were analyzed with the Kaplan and Meier method [18]. All reported P values were generated from a two-sided analysis via the SPSS 11.0 system (SPSS, Inc, Chicago, IL).

## Results

### Patient characteristics

Between May 2004 and February 2006, a total of 33 consecutive patients who obtained PR or MR after last chemotherapy from 106 NSCLC patients entered the study. Characteristics of the eligible patients are listed in Table 1. All 33 patients were evaluable for both response and toxicity. The median age was 58 years (range from 31 to 72). Most of the patients were male (66.7%). Ten patients (30.3%) had stage IIIb disease, and 23 (69.7%) had stage IV disease at study entry. Six patients (18.2%) had PS ≥ 2, and 23 patients (69.7%) had at least one measurable metastatic lesion. Thirteen (39.4%) had failed one prior chemotherapy regimen; 8(24.2%) failed to respond to at least two prior regimens; all the 21 pretreated patients had failed to at least one line of platinum-based chemotherapy; only 12(36.4%) patients had been chemotherapy-naïve prior to the most recent chemotherapy regimen. Before gefitinib, 6 (18.2%) received docetaxel alone, the other 27 (81.8%) received platinum-based regimen. Twenty-six patients (78.8%) achieved PRs, 7 (21.2%) achieved MRs after 2 or 3 cycles of chemotherapy. Five patients (15.2%) had undergone prior surgery with curative intent. Fourteen (42.4%) of them were smokers. Most of the non-smokers were female. Twenty-three had adenocarcinomas (69.7%) and 14 of them were male patients.

### Efficacy and survival

Gefitinib was administered, with a median treatment duration of 6.5 months (95%CI, 1 to 16.6 months). The overall RR was 24.2% (8 of 33) (95% CI, 11% to 42%). There were 7 PRs (21.2%) and one CR (3%). Stable disease was recorded in 69.7% (23 patients) and progressive disease in 6.1 % (2 patients) (Table 2). The overall disease control rate, including CR, PR and SD, was 93.9%. A comparable response rate was observed for adenocarcinoma and squamous carcinoma patients as 8 of the 24 patients with adenocarcinoma had a major objective response (33.3%), while none of the 9 patients with squamous carcinoma had a major objective response. Rapid tumor regression was demonstrated for most of the responders. Among these responders, 7 patients (87.5%) obtained an objective response as measured by the first post-baseline assessment. One patient met the response criteria in the second month. Responses, including partial and/or complete responses, were observed at all sites of disease such as primary tumor, liver, and lymph node metastases.

**Figure 1:**
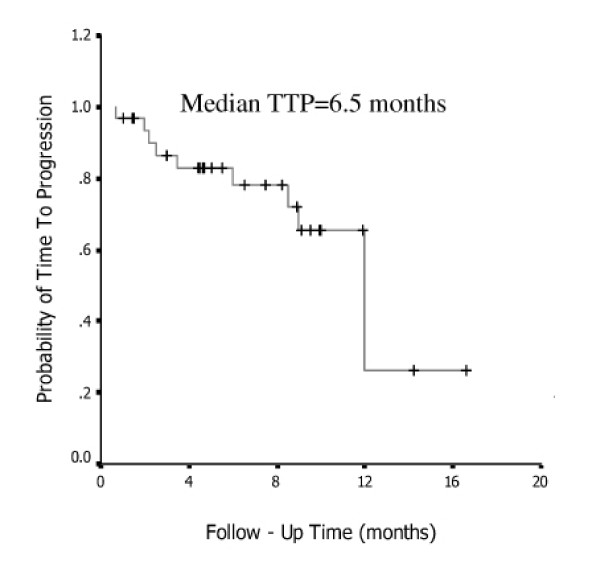
Time to progression of 33 patients treated with gefitinib.

**Figure 2:**
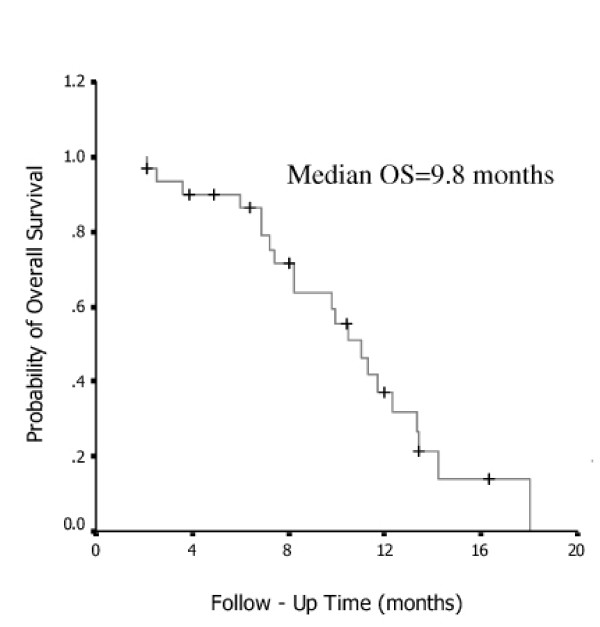
Overall survival of 33 patients treated with gefitinib.

The median duration of response was 7 months (95%CI, 4.0 to 13.2 months); the median time to disease progression was 6.5 months (95%CI, 0.7 to 16.6 months) (Fig 1), the median OS was 9.8 months (range, 2.1 to 18.0 months) (Fig 2). Eleven patients (33.3%) were dead after a median follow-up of 10 months (95%CI, 2.1 to 18.0 months). The actual 1-year survival rate was 36.4%. There was no significant difference in the response rate, duration of response, or OS according to performance status. However, patients with stage IIIb disease had a tendency toward longer OS compared to those with stage IV disease (P = 0.061).

### Disease-related symptom improvement and toxicity

All 33 patients were assessable for toxicity and symptom improvement. Improvements in disease-related symptoms are shown in Table 3. The symptom improvement rate was 54.5% (18 of 33)(95% CI, 41% to 69%) in patients with a tumor response, and 36.3% (95% CI, 20%–55%) in patients without a response. The difference was not statistically significant (χ^2 ^test: P = 0.182). Although AEs existed in this study, therapy was well tolerated. Toxicity was mainly mild (CTC. G1 or G2). G3 and G4 toxicity was uncommon, and most events were reversible (Table 4). Drug-related AEs were observed in 30.3% of patients. Only grade 3 and grade 4 AEs required a short treatment interruption, and none required a dose reduction. No drug-related deaths occurred. Twenty-three patients (69.7%) were either asymptomatic or showed no worsening of their symptoms during the study. The severe drug-related G3 or G4 AEs including diarrhea, vomiting and nausea only occurred in one patient who had to be hospitalized for severe diarrhea. None of the patients was withdrawn from the trial due to AEs.

## Discussion

This paper describes the first phase II trial of sequential administration of gefitinib following tumor responses to chemotherapy in patients with advanced NSCLC. The TTP and OS observed are encouraging, although patient selection bias cannot be excluded. It is unclear whether having achieved a response from chemotherapy influences the efficacy of subsequent gefitinib. It should be noted that the TTP and OS registered in this study are comparable to historical data obtained in patients who received first-line chemotherapy for six cycles [4,19,20]. Although chemotherapy had administered two or three cycles at the physician's discretion, the TTP in this study is comparable to the duration of response of continuous chemotherapy reported in the literature. Furthermore, the calculations of OS in continuous chemotherapy and in sequential gefitinib are both from the start of responding chemotherapy until death.

To date, second-line chemotherapy has only shown modest efficacy in advanced NSCLC with overall RRs ranging from 6% to 25%, median duration of response ranging from 1.2 months to 7.5 months, and median OS ranging from 3.2 months to 9.5 months [4,20,22-24]. Previous studies indicated that second-line chemotherapy mainly plays a palliation role in advanced NSCLC. The QOL improvement in chemotherapy is mainly dependent on its efficacy. Patients with advanced NSCLC usually have an extremely poor prognosis and exhibit severe symptoms if their tumor progresses after chemotherapy. Therefore, this trial sought to increase or maintain the efficacy of chemotherapy by adding gefitinib after tumors responded to chemotherapy.

Some evidence had demonstrating that chemosensitivity is mainly related to the proliferative activity of tumor cells and reductions of the proliferation rate are associated with chemoresistance [12,25]. In this regard, chemotherapy is more effective in tumors with higher proliferative rates. The clinical efficacy of chemotherapy could be influenced by the simultaneous administration of gefitinib, which leads to a decrease in growth factor signaling. Furthermore, our previous *in vitro *studies reported that the sequential administration of EGFR inhibitors after chemotherapy may enhance/maintain chemotherapy-induced cancer cell damage [26,27]. By contrast, subsequent gefitinib will not be expected to achieve its potential synergy with chemotherapy if a tumor did not respond to chemotherapy. Considering that the combination effect is schedule-dependent, the rationale would be to administer the gefitinib when chemotherapy is inducing or has induced maximum tumor damage. Thus, in the present trial, only patients with an MR or PR to chemotherapy were included. Although one patient (3%) achieved a CR, the sequential administration of gefitinib following a minor or partial response to chemotherapy did not appear to result in a greater RR than with single agent gefitinib. The TTP and OS achieved using this sequential administration strategy were comparable to those attained with combination chemotherapy regimens including paclitaxel plus cisplatin (PC), gemcitibine plus cisplatin (GC) or navelbine plus cisplatin (NP).

It has been reported that the overall RRs of PC, GC and NP regimens are 30% to 40% in chemotherapy-naïve patients, with CRs of less than 5%. The RR was lower in second-line settings, varying from 6% to 21% [28,29]. Clinical study demonstrated that a significant proportion of patients with advanced NSCLC have subjective symptoms, such as anorexia, weight loss, and impairment of performance status [28]. Since QOL is a major end point of second-line chemotherapy, severe toxicity is not acceptable in this setting. However, grades 3 and 4 toxicities were frequently reported in the PC, GC or NP regimens [3,19]. Based on our clinical experience, response is usually apparent after the initial two to three cycles of chemotherapy. This means that subsequent chemotherapy of three to four cycles, 2.3 to 3.0 months, is only to consolidate the clinical response obtained. These regimens yield median duration of response of 4 to 6 months in advanced patients who have thus been experiencing chemotherapy with about 50% to 70% median duration of response and only 30% remission time free of chemotherapy and its associated toxicity.

## Conclusion

Although the optimal schedule of gefitinib in advanced NSCLC has not yet been defined, the present study suggests that sequential administration of gefitinib following chemotherapy is a promising approach in the management of advanced NSCLC. A randomized trial to identify this sequential schedule of administration of gefitinib and chemotherapy is required to ensure better clinical efficacy in the treatment of advanced NSCLC.

## Competing interests

The author(s) declare that they have no competing interests.

## Authors' contributions

JMX designed the experiments and wrote the manuscript. YH, YML, CHZ and YW participated in patients' follow-up and tumor response evaluation. AP participated in the data analysis. All authors read and approved the final manuscript.

## Pre-publication history

The pre-publication history for this paper can be accessed here:



## Figures and Tables

**Table 1:** Characteristics of the patients

Characteristic	Value
Age, years	
Median	58
Range	31–72
Sex	
Male	22(66.7)
Female	11(33.3)
ECOG performance status	
0	13(39.4)
1	14(42.4)
2	5(15.2)
3	1(3.0)
Smoking History	
Smoker	14(42.4)
Non-smoker*	19(57.6)
Histology	
Adenocarcinoma	24(72.7)
Squamous cell carcinoma	9(27.3)
Stage at study entry	
III B	10(30.3)
IV	23(69.7)
No. of Prior failed chemotherapy regimen	
0	12(36.4)
1	13(39.4)
≥2	8(24.2)
Chemotherapy response prior to gefitinib	
MR	7(21.2%)
PR	26(78.8%)

*Non-smoker = patients who had smoked less than 100 cigarettes in their lifetime.

**Table 2:** Response in 33 Assessable Patients

Response	All Patients	
	
	No.	%
Complete response	1	3
Partial response	7	21.2
Stable disease	23	69.7
Progressive disease	2	6.1
Total	33	100

**Table 3:** Symptom Improvement in 33 Assessable Patients

Response	Symptom Improvement	
	No.	%

CR+PR	1+5	18.2
SD	12	36.3
PD	0	0

Total	18	54.5

**Table 4:** Drug-Related Adverse Events in 33 Assessable Patients

Adverse Event	Grade 1		Grade 2		Grade 3		Grade 4	
				
	No.	%	No.	%	No.	%	No.	%
Skin								
Rash	3	9.1	3	9.1	1	3.0	0	0
Pruritus	2	6.1	2	6.1	0	0	0	0
Dry skin	1	3.0	0	0	0	0	0	0
Acne	1	3.0	1	3.0	0	0	0	0
Digestive								
Diarrhea	5	15.2	1	3.0	0	0	1	3.0
Nausea	2	6.1	1	3.0	1	3.0	0	0
Vomiting	0	0	0	0	1	3.0	0	0
Anorexia	2	6.1	0	0	0	0	0	0
Costiveness	1	3.0	0	0	0	0	0	0
Metabolic								
Increased ALT	1	3.0	0	0	0	0	0	0
Increased AST	1	3.0	0	0	0	0	0	0
